# Transcriptomic Analysis of MAPK Signaling in NSC-34 Motor Neurons Treated with Vitamin E

**DOI:** 10.3390/nu11051081

**Published:** 2019-05-15

**Authors:** Luigi Chiricosta, Agnese Gugliandolo, Giuseppe Tardiolo, Placido Bramanti, Emanuela Mazzon

**Affiliations:** IRCCS Centro Neurolesi “Bonino-Pulejo”, 98124 Messina, Italy; luigi.chiricosta@irccsme.it (L.C.); agnese.gugliandolo@irccsme.it (A.G.); giuseppe.tardiolo@irccsme.it (G.T.); placido.bramanti@irccsme.it (P.B.)

**Keywords:** vitamin E, α-tocopherol, motor neuron, amyotrophic lateral sclerosis, MAP kinases

## Abstract

Vitamin E family is composed of different tocopherols and tocotrienols that are well-known as antioxidants but that exert also non-antioxidant effects. Oxidative stress may be involved in the progression of neurodegenerative disorders including amyotrophic lateral sclerosis (ALS), characterized by motor neuron death. The aim of the study was the evaluation of the changes induced in the transcriptional profile of NSC-34 motor neurons treated with α-tocopherol. In particular, cells were treated for 24 h with 10 µM α-tocopherol, RNA was extracted and transcriptomic analysis was performed using Next Generation Sequencing. Vitamin E treatment modulated MAPK signaling pathway. The evaluation revealed that 34 and 12 genes, respectively belonging to “Classical MAP kinase pathway” and “JNK and p38 MAP kinase pathway”, were involved. In particular, a downregulation of the genes encoding for p38 (Log_2_ fold change −0.87 and −0.67) and JNK (Log_2_ fold change −0.16) was found. On the contrary, the gene encoding for ERK showed a higher expression in cells treated with vitamin E (Log_2_ fold change 0.30). Since p38 and JNK seem more involved in cell death, while ERK in cell survival, the data suggested that vitamin E treatment may exert a protective role in NSC-34 motor neurons. Moreover, Vitamin E treatment reduced the expression of the genes which encode proteins involved in mitophagy. These results indicate that vitamin E may be an efficacious therapy in preventing motor neuron death, opening new strategies for those diseases that involve motor neurons, including ALS.

## 1. Introduction

The vitamin E family includes eight isomers divided into tocopherols and tocotrienols. The number and the localization of the methyl groups on the chromanol rings differentiated the isomeric forms of tocopherols and tocotrienols, that are respectively, α-, β-, γ-, and δ-tocopherol and α-, β-, γ-, and δ-tocotrienol. The antioxidant action of vitamin E is the best known and characterized. Alpha-tocopherol is present in many edible oils while its content in fruits and vegetables with low lipid content is normally negligible [1].

The α-tocopherol, that represents the major form of vitamin E in tissues, is well known for its antioxidant action but it is also able to exert gene regulation properties [2]. Indeed, vitamin E can exert its action through both redox-dependent and independent mechanisms, modulating enzymes and receptors involved in signal transduction and gene expression pathways also affecting the action of different transcription factors [3]. The modulation of signal transduction pathways seems to be also mediated in a non-antioxidant manner. In particular, vitamin E can modulate pathways involved in cell death, survival, and regeneration [3]. Moreover, both in vitro and in vivo evidence indicated a regulatory potential of vitamin E metabolites, in particular in inflammatory processes [4].

Vitamin E plays also a pivotal role for neurons. Indeed, vitamin E deficiency was reported to cause cellular atrophy and a reduction of dendritic branching of Purkinje neurons together with cognitive deficits, while vitamin E supplementation prevented these defects, indicating that an adequate vitamin E concentration is necessary for the physiological function of Purkinje neurons [5]. Vitamin E deficits are associated with impaired motor coordination, cognitive functions, ataxia, and lipid peroxidation [5,6]. Moreover, vitamin E deficiency may cause an impairment of the blood–brain barrier through the increase of brain oxidative stress [7].

Among the different processes that take part in the development of neurodegeneration, also oxidative stress may play a role in the progression of neurodegenerative diseases, characterized by neuron loss associated with compromised motor or cognitive functions. Interestingly, neuronal cells are particularly sensitive to oxidative damage because of their high polyunsaturated fatty acid content in membranes and elevated oxygen consumption [8,9]. For this reason, antioxidant therapies, including vitamin E, received attention as promising approaches for neurodegenerative disorders, however, the results are not clear yet [10].

Vitamin E supplementation was highlighted by some reports to play a beneficial action in preclinical and clinical studies of neurodegeneration, such as Alzheimer’s disease [11] and Parkinson’s disease [12], even if vitamin E effects in patients are still under debate. Indeed, results in vitro and in animal models seem promising, but clinical trials in patients affected by neurodegenerative disorders showed contrasting results.

The situation is similar for amyotrophic lateral sclerosis (ALS). A study evidenced that the vitamin E supplementation in a transgenic ALS model delayed disease onset and slowed progression, but did not prolong survival [13]. An increase of oxidative stress, indicated by altered levels of oxidative stress markers, was found also in ALS patients [14], and interestingly, some studies evaluated whether vitamin E may exert beneficial actions in ALS patients with contrasting results. Higher baseline serum α-tocopherol levels were associated with a lower ALS risk, but α-tocopherol supplementation did not show significant effects on ALS risk [15]. A study suggested that vitamin E supplementation may be used to prevent ALS. In particular, ALS rates declined with increasing years of use, highlighting that long-term vitamin E supplement was associated with lower ALS rates [16]. Moreover, patients receiving riluzole plus α-tocopherol were less likely to progress from the milder state to the more severe state of the ALS Health State scale, even if α-tocopherol did not show effects on the survival and the motor function [17]. On the contrary, another work evidenced no difference in the quality of life of ALS patients receiving or not vitamin E supplementation [18]. Then, other clinical trials are needed to clarify the real efficacy of vitamin E administration in ALS patients, with a careful choice of the patients, because it is also possible that only some of them, with particular features, may benefit of vitamin E administration.

The aim of this study was to evaluate the transcriptional modifications induced by vitamin E treatment in NSC-34 motor neurons using Next Generation Sequencing (NGS) analysis, focusing on the pathways modulated by vitamin E treatment in order to study its potential beneficial effects in diseases that involve motor neurons.

## 2. Materials and Methods

### 2.1. Cell Culture and Vitamin E Treatment

For this study, the NSC-34 motor neuron cell line was used. NSC-34 cells are considered the most stable motor neuron cell line model and are widely used, also because they present properties specific of motor neurons. For this reason, they were used as a cellular model to study the pathophysiology of ALS [19]. NSC-34 cells were obtained from Cellutions Biosystems Inc., Cedarlane (Burlington, ON, Canada) and were cultured in DMEM-high glucose medium (Sigma-Aldrich, Saint Louis, MO, USA) supplemented with 10% FBS (Sigma-Aldrich), at 37 °C in a moisturized atmosphere of 5% CO_2_ and 95% air. In order to evaluate the effects of vitamin E treatment, NSC-34 cells were treated for 24 h with 10 µM vitamin E (VitE-NSC-34), in the form of α-tocopherol acetate. At the end of the treatment, cells were harvested for RNA extraction.

### 2.2. Thiazolyl Blue Tetrazolium Bromide (MTT) Assay

The effects of the treatment with vitamin E on cell viability were evaluated using the MTT assay. NSC-34 cells were cultured in 96-well plates and incubated for 24 h with different vitamin E concentrations (1, 5, 10, 15, 20 µM). At the end of the treatment, cells were washed and incubated with fresh medium containing MTT (0.5 mg/mL; Sigma-Aldrich) at 37 °C for 4 h. After, formazan crystals were dissolved in acidic isopropanol at 37 °C for 1 h and the optical density was evaluated by spectrophotometric measurement of absorbance. The assay was repeated for three independent times, each time in triplicate (in total 9 replicates). The aim of the study was evaluating the effects of vitamin E in NSC-34 motor neurons. For this reason, the choice of the vitamin E concentration for transcriptomic analysis was based on MTT assay and previous literature data indicating that 10 µM vitamin E was able to decrease oxidative stress and to prevent cytotoxicity in different in vitro experiments, proving to be more efficient than higher doses [20,21].

### 2.3. Statistical Validation of Cell Viability

The results are expressed by mean ± SD. Statistical analysis of cell viability was performed using GraphPad Prism version 7.0 software (GraphPad Software, La Jolla, CA, USA). In order to test the normality of the data, the Shapiro–Wilk normality test was performed. All the groups appeared normally distributed against a *p* value of 0.05. The multiple comparison was carried out using one-way ANOVA test and Bonferroni post hoc test. A *p* value less than or equal to 0.05 was considered statistically significant.

### 2.4. Extraction of Total RNA and cDNA Library Preparation

RNA was obtained using the Maxwell^®^ RSC simplyRNA Cells Kit (Promega) following the manufacturer’s instruction. The library preparation was carried out according to the TruSeq RNA Access library Prep kit protocol (Illumina, San Diego, CA, USA) using the same protocol described by Chiricosta et al. [22].

### 2.5. RNA-Seq Data Analysis and Gene Evaluation

The MiSeq NGS instrument was used to perform the cDNA analysis, in parallel, both for treated and untreated samples. The platform provided “bcl” format files for the multiplexed samples. Those files were demultiplexed in “Fastq” format files by CASAVA software (version 1.8). RNA-seq data analysis was performed following the best practices illustrated by Conesa et al. [23]. The quality check analysis of the Fastq files was performed by fastQC software. A first preprocessing of the reads was made by Trimmomatic in order to remove adapters and low quality bases (LEADING:30 TRAILING:30 SLIDINGWINDOW:4:28 MINLEN:35). The reads were then aligned against the “Mus musculus” reference genome provided by University of California Santa Cruz (UCSC) website (http://labshare.cshl.edu/shares/gingeraslab/www-data/dobin/STAR/STARgenomes/ENSEMBL/mus_musculus/ENSEMBL.mus_musculus.release-75/) and sorted by STAR RNA-seq aligner. The Cuffdiff software version 2.2.1 was used to find changes in the expression of the transcripts among the samples and to associate each transcript to the related genes taking advantage of Mus musculus GTF file provided still by UCSC website. The information of the MAPK signaling pathway and Mitophagy pathway were extracted from the KEGG database (https://www.genome.jp/kegg/pathway.html) [24] and the genes in the obtained set that were included in these pathways were searched for.

## 3. Results

### 3.1. Cell Viability

In order to assess the cytotoxic effects of vitamin E, NSC-34 motor neurons were exposed to different concentrations of vitamin E and the cell viability was evaluated. Vitamin E in the range of concentrations between 1 µM to 20 µM did not show cytotoxicity, as demonstrated by MTT assay. Indeed, the percentage of viable cells was pretty similar to the control cells (CTR-NSC-34) in all groups. Since the viability data follow the normal distribution according to Shapiro–Wilk normality test, the statistical analysis was performed using ANOVA and it confirmed no significant variation between control and treated cells (*p* value > 0.99 for each comparison) (Table 1).

### 3.2. Transcriptome Analysis and Gene Inspection

The RNA-Seq analysis performed by Cuffdiff software reveals 8556 genes among which 3064 genes statistically differ in their expression (*q* value < 0.05). It was investigated which of the statistically significant genes take part in “MAPK signaling pathway” (map04010) provided by KEGG database. Among the subpathway described in KEGG, “Classical MAP kinase pathway” and “JNK and p38 MAP kinase pathway” were evaluated. In particular, 34 genes were included in “Classical MAP kinase pathway” (Table 2) while 12 genes were inside “JNK and p38 MAP kinase pathway” (Table 3).

**Figure 1:**
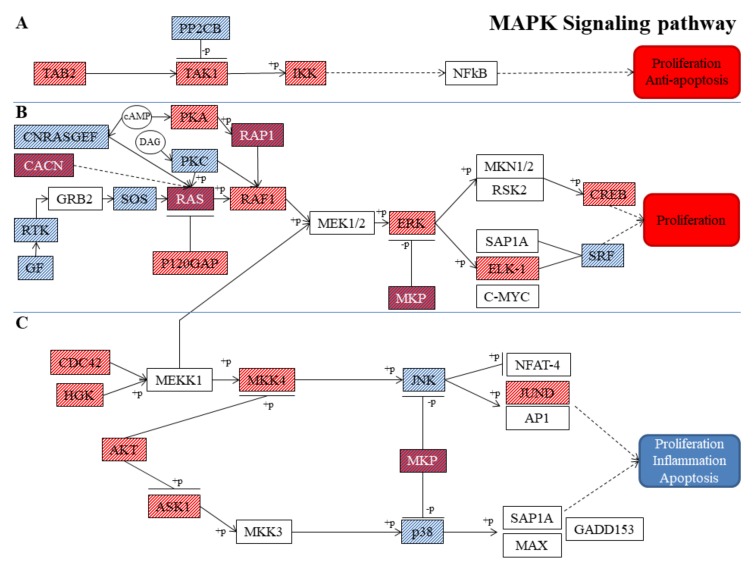
Set of motor neuron proteins in the MAPK signaling pathway. Classical MAP kinase pathway not mediated (**A**) and mediated (**B**) by ERK protein. JNK and p38 MAP kinase pathway mediated by JNK and p38 proteins (**C**). Red background represented proteins encoded by upregulated genes in VitE-NSC-34. Blue background represented proteins encoded by downregulated genes in VitE-NSC-34. The blue and red mixed background indicated that the different genes encoding those proteins are in part upregulated and in part downregulated in VitE-NSC-34. The white background indicated that there is no statistical difference in the level of gene expression. The dotted lines highlight indirectly modulations, the complete lines show directly modulations.

Their distribution is highlighted in Table 4. 16 genes belonging to “Classical MAP kinase pathway” were upregulated in VitE-NSC-34, while 18 genes were downregulated (Table 4). Looking at the “JNK and p38 MAP kinase pathway” 8 genes were upregulated in VitE-NSC-34 and 4 genes were downregulated in VitE-NSC-34 (Table 4). The pathway in which the genes are involved is described in Figure 1.

The “Mitophagy-Animal KEGG pathway” (map04137) was also investigated. In this pathway, 13 genes were differentially expressed between VitE-NSC-34 and CTR-NSC-34 (Table 5). In particular, 4 genes were upregulated in VitE-NSC-34 and 9 genes were downregulated in VitE-NSC-34.

## 4. Discussion

Motor neuron degeneration is the main characteristic of ALS. The etiopathology of ALS disease is not clear yet. The most known causes of motor neuron death are linked to oxidative stress, excitotoxicity, protein aggregation [14]. In particular, neuronal cells are particularly sensitive to oxidative stress damage [9]. Anti-oxidant therapies may be helpful in order to limit neuronal cell death. Many antioxidants, including vitamin E were tested to improve neurodegenerative disorders. However, even if in vitro results seem interesting, in vivo data reported contrasting results, given that only some studies showed real improvements, while other reports indicated a failure. Moreover, even if vitamin E has well known antioxidant functions, it is also able to exert non-antioxidant actions modulating gene expression and signaling pathways that may exert a protective action.

In this work, vitamin E was able to modulate MAPK pathway. The MAPK pathway controlled different cell processes, including proliferation, cell survival and cell death [25]. In particular, it is reported that JNK and p38 seem to play a role in cell death, including in neuronal cell degeneration and apoptosis even in ALS, while ERK seems to induce cell survival [25].

In this study, both *MAPK9*, encoding for JNK, and *MAPK11* and *MAPK14*, encoding for p38, were downregulated in VitE-NSC-34.

The JNK pathway consists of JNK, a MAP2K such as MKK4, and a MAP3K such as ASK1 and MEKK1 [25]. According to KEGG, also CDC42 and HGK take part in the activation of the JNK pathway. *CDC42* and *MAP4K4* genes, upregulated in VitE-NSC-34, encode respectively for the GTPase CDC42 and for the kinase HGK. Interestingly, CDC42 belongs to a family of Rho GTPases, which also play a role in neuronal survival [26]. MEKK1 does not significantly change its expression level. It is important to notice that MEKK1 protein can also phosphorylate MEK1/2 complex in the ERK pathway [27], then the upregulation of *CDC42* and *MAP4K4* genes may be due to the activation of ERK pathway. MKK4 is encoded by *MAP2K4* gene that more expressed in VitE-NSC-34.

The p38 pathway includes different MAP2Ks, while MAP3Ks are in part in common with the JNK pathway, such as ASK1. However, *MAP3K5* was upregulated in VitE-NSC-34 and its protein product ASK1 phosphorylates MKK3. ASK1 has a role in neurite outgrowth in NSC-34 motor neuronal cells through the regulation of survival motor neuron (SMN) protein level [28].

Even if the genes encoding for ASK1 as well as MKK4 were upregulated in VitE-NSC-34 also the genes encoding for their negative modulator, the kinase AKT, encoded by *AKT1* and *AKT3* genes, were upregulated in VitE-NSC-34. *AKT3* is able to exert neuroprotective effects in spinal cord motor neurons in vitro and in a mouse model of ALS [29]. Then, its upregulation may be beneficial in this experimental condition, exerting a protective action in NSC-34 motor neurons. 

The final products of the JNK and p38 mediated MAPK Signaling pathway are complex of transcription factors among which *JUND* gene, encoding for JunD protein, resulted more expressed in VitE-NSC-34. Interestingly, JunD seems to be involved in differentiation, proliferation and apoptosis. Moreover, JunD is also regulated by ERK [30], that is upregulated in NSC-34 cells treated with vitamin E. These results indicated that vitamin E treatment was able to induce a protective response in NSC-34 motor neurons reducing the expression of p38 and JNK. However, some genes participating in these pathways showed an upregulated expression. Interestingly, these genes, such as *CDC42, MAP3K5*, and *AKT3*, are involved in important functions for neuronal cells as already said.

Vitamin E increased the expression of *MAPK1* that encoded for ERK. In the nervous system, ERK modulates synaptic plasticity, brain development and it is involved in different neurodegenerative diseases [27]. For these reasons the modulation of ERK pathway in motor neurons by vitamin E is really interesting and allows to deepen the knowledge about vitamin E effects. Indeed, ERK activation seems to exert a protective action in ALS models [31]. In the ERK pathway, ERK is activated by MEK1/2, that in turn is regulated by MAP3Ks, the Raf proteins, that are activated by Ras [25]. In this study, the genes encoding for ERK, Raf, NRAS, and KRAS were upregulated in VitE-NSC-34, while MEK1/2 expression did not change significantly. Only HRAS was downregulated but with a low fold change. Increased intraneuronal Ras signaling is considered generally neuroprotective [32]. Ras activity, and then ERK pathway, can be regulated by different stimuli, such as calcium [33], growth factors or cyclic adenosine monophosphate (cAMP).

Calcium is a second messenger and in neurons, it regulates differentiation, synaptic transmission, and plasticity [34]. Alterations of calcium concentrations are associated with neuronal death and then to different neurodegenerative diseases [34]. AMPA receptors were involved in the excitotoxicity of motor neurons in ALS [35]. In particular, oxidative stress is involved in the disruption of Ca^2+^ homeostasis in neuronal cells and in their death and interestingly, Ca^2+^ overload is a possible trigger of motor neuron death in ALS [35]. Moreover, another type of channels, the voltage-gated Ca^2+^ channels (VGCCs) showed an aberrant function and localization in ALS mouse motor neurons [36]. A study demonstrated that AMPA induced the influx of Ca^2+^ also through L-type VGCCs [37] and their inhibition may protect against degeneration of motor neurons. The CTR-NSC-34 expressed eight VGCCs of which one (*CACNA1H*) belongs to the T-type (subunit α1, also known as Ca_v_3.2), and seven (*CACNA1C* encoding for subunit Ca_v_1.2, *CACNA2D1, CACNA2D2* encoding for subunits α2δ, *CACNB1* encoding for subunit β, *CACNG2, CACNG3, CACNG7* encoding for subunits γ) to the L-type. Among them, just the subunit *CACNG3* and *CACNG7* of the L-type gamma were overexpressed in VitE-NSC-34, therefore calcium inside VitE-NSC-34 may be reduced, with the consequent protection of motor neurons.

Also, growth factors such as BDNF can activate Ras through the recruitment of a complex formed by GRB2 and SOS leading to the activation of the Raf/MEK/ERK cascade as evidenced by KEGG. Except for GRB2, expressed in CTR-NSC-34 but without statistically change in the level of expression compared to VitE-NSC-34, all the other proteins are encoded by genes downregulated in VitE-NSC-34 (*BDNF*, *VEGFA*, *ERBB3*, *SOS1*, *SOS2*). These data are in line with a study that showed that vitamin E was able to protect cortical neurons from oxidative stress through the activation of MAPK pathway, but BDNF signaling is not involved in the vitamin E effects, indeed neither BDNF expression nor TrkB receptor activity were influenced by vitamin E treatment [38].

The secondary messenger cAMP is able to activate the ERK pathway through Protein Kinase A (PKA), that activate Raf through Rap1, or cAMP can activate RAS through the Guanine Nucleotide Exchange Factor (CNRASGEF). In this work, *PRKACB,* encoding for PKA and *RAP1B*, were upregulated in VitE-NSC-34. Only *RAP1A* and *RAPGEF2*, encoding for CNRASGEF, were downregulated. The cAMP/PKA signaling is known to regulate mitochondria functions, and alterations of this pathway exert harmful effects in different pathological conditions, such as neurodegenerative disorders [39]. Then, all together, these results may indicate that vitamin E treatment in motor neurons NSC-34 may activate the ERK pathway through the activation of cAMP/PKA. Accordingly, it was demonstrated that vitamin E can induce cAMP production [40]. 

Ras can be activated through the Protein Kinase C (PKC). However, PKC overexpression may be pathogenic for ALS [41]. The *PRKCB* gene expression, encoding for one of the PKC, resulted to be reduced in VitE-NSC-34. Vitamin E, and in particular the isoform α-tocopherol, induced PKC inhibition [2,3].

When ERK is activated, it acts downstream to activate several transcription factors, like Elk-1 encoded by the *ELK1* gene that was upregulated in VitE-NSC-34. Elk-1 regulates immediate early gene expression associated with a dimer of serum response factor (SRF), that was downregulated in this experiment. Its function in neurons is not completely clear, and it depends on its localization. However, when it is activated through phosphorylation by MAPK/ERK, Elk-1 moves to the nucleus where it regulates chromatin remodeling and neuronal differentiation [42].

ERK activation leads also to the activation of the transcription factor CREB. The ATF4 protein, belonging to CREB family, is encoded by *ATF4* gene that was upregulated in this experiment [43]. ATF4 was shown to be needed for normal synaptic plasticity and memory in vitro and in vivo [44]. Interestingly, a work evidenced that α-Tocopherol protects renal cells from oxidative stress activating CREB through ERK and PKA [45].

The activation of ERK is finely regulated by protein phosphatase, that dephosphorylating ERK, create a negative feedback needed to modulate Ras⁄Raf⁄ERK signaling. Indeed, the expression of some MAPK phosphatases (MKP) as response to growth factor and mitogen stimulation is mediated by ERK [46]. *DUSP1*, *DUSP4* and *DUSP16* genes encode for MKP proteins. *DUSP4* was downregulated in VitE-NSC-34, while both *DUSP1* and *DUSP16* were upregulated. Moreover, DUSP1 and DUSP16 show also neuroprotective actions [47,48]. The same MKP also contributes to the inhibition of JNK and p38. Furthermore, Ras is negatively modulated by P120GAP, encoded by *RASA1* that is more expressed in VitE-NSC-34.

The results of this study evidenced vitamin E capacity to influence the gene expression of the genes involved in MAPK pathway. Interestingly, vitamin E induced the selective upregulation of ERK associated with the upregulation of PKA. On the contrary, the genes involved in the other pathways leading to ERK activation, including the increase of calcium and PKC that play a negative role in neuronal cells and also in ALS, showed a decreased expression.

The physiological cellular proliferation in the classical MAPK Signaling pathway may be also mediated by the transcription factor NF-κB that promotes neuron survival [49]. Interestingly, the results evidenced that vitamin E modulated also this pathway. TAK1 protein, encoded by *MAP3K7* gene, indirectly acts on the activation of NF-κB. TAK1 assembly with TAK1 binding protein 2 (TAB2) and, after the activation, induces the activation of IκB kinase (IKK) [50]. The IKK protein phosphorylates the inhibitor of NF-κB, inducing its dissociation and then the activation of NF-κB. The genes *MAP3K7, TAB2* and *IKBKB,* encoding for the subunit β of IKK, were upregulated in VitE-NSC-34. Conversely, the *PPM1B* gene encodes for the phosphatase PP2CB and it was downregulated in VitE-NSC-34. TAK1 can be activated also HGK, that is involved in JNK pathway [50].

JNK is known to regulate mitophagy [51]. According to the downregulation of the gene encoding for JNK, even the main genes involved in the mitophagy pathway were downregulated in VitE-NSC-34. This result is in accordance also with the upregulation of PKA. Indeed, cAMP/PKA is reported to inhibit mitophagy [39]. Mitophagy may be activated by different triggers, including oxidative stress, and it is associated with neurodegenerative diseases, such as ALS [52]. The only genes upregulated in VitE-NSC-34 involved in mitophagy were *ATF4*, *USP15*, *SQSTM1*, and *GABARAPL1,* encoding respectively for the proteins ATF4, USP15, p62 and GABARAPL1, member of the LC3/GABARAP family [53]. USP15 is a deubiquitinating enzyme that limits Parkin-mediated mitophagy [54]. ATF4, p62 and LC3 play important roles in neuronal cells other than exerting their action in mitophagy and it is possible that their upregulation was associated with the neuronal function. ATF4 influences GABA_B_R trafficking and, in turn, neuronal plasticity [55]. On the other hand, p62 is essential for neuronal differentiation [56]. Moreover, the loss of p62 caused a shorter lifespan and a decline in motor function in a SOD1 ALS mouse model [57]. GABARAPL1 was expressed in the brain since embryonic day 11 and progressively increases in the adult [58]. Then, the results highlight vitamin E capacity to downregulate the genes involved in the mitophagic process, probably through the downregulation of JNK and the upregulation of PKA.

However, one limitation of the study is that vitamin E effects were investigated in vitro in the motor neuron cell line NSC-34. The evaluation of vitamin E actions in primary motor neurons or in NSC-34 cells expressing mutant SOD1 as ALS model would be also interesting. Moreover, also the study of the potential vitamin E protective action in motor neurons exposed to inflammatory or pro-oxidant stimuli and in vivo in murine models of ALS would be helpful to deepen the knowledge on vitamin E effects and on the pathways that it can modulate. Also, the evaluation of the effects of other vitamin E isomers would be interesting in order to verify if the different compounds have the same or different actions.

## 5. Conclusions

The results of this study indicated that Vitamin E treatment may exert a protective action in motor neurons. Indeed, transcriptomic analysis revealed the upregulation of ERK, while p38 and JNK, together with the genes involved in the mitophagy pathway, were downregulated. In particular, ERK activation could be mediated by cAMP/PKA. Then, these results indicated that vitamin E, inducing a protective response, may be a promising therapeutic strategy in those conditions where motor neuron death is present, such as ALS. Moreover, this work allows us to increase the knowledge about vitamin E modulation of signaling pathways in neuronal cells.

## Figures and Tables

**Table 1:** Cell viability. The table shows mean and standard deviation of each group and the *p* value of the Shapiro–Wilk normality test. The values are rounded to the second decimal digit.

Experimental Group	Mean (%)	Standard Deviation	*p* Value
Control	100.00	10.10	0.12
1 µM Vit. E	98.30	3.62	0.18
5 µM Vit. E	100.10	6.12	0.67
10 µM Vit. E	99.14	7.88	0.31
15 µM Vit. E	97.87	9.86	0.24
20 µM Vit. E	98.84	8.53	0.66

**Table 2:** Statistically significant genes involved in KEGG classical MAP kinase pathway, with their expression levels in CTR-NSC-34 and VitE-NSC-34, the Fold change computed as the Log_2_(VitE-NSC-34—CTR-NSC-34), the q value and the name of the gene product in KEGG. The values are rounded to the second decimal digit.

Gene	Expression Level CTR-NSC-34	Expression Level VitE-NSC-34	Fold Change	*q* Value	KEGG
*CACNA1H*	19.33	8.34	−1.21	6.06 × 10^−3^	CACN
*CACNG3*	29.49	46.23	0.65	9.75 × 10^−4^	CACN
*CACNG7*	9.53	15.87	0.74	3.69 × 10^−4^	CACN
*CACNA1C*	27.86	23.16	−0.27	3.69 × 10^−4^	CACN
*CACNA2D1*	24.54	20.98	−0.23	2.34 × 10^−2^	CACN
*CACNA2D2*	4.75	2.11	−1.17	1.98 × 10^−2^	CACN
*CACNB1*	27.88	15.59	-0.84	3.69 × 10^−4^	CACN
*CACNG2*	8.68	5.00	−0.80	3.69 × 10^−4^	CACN
*BDNF*	5.44	2.03	−1.42	3.69 × 10^−4^	GF
*VEGFA*	519.96	446.13	−0.22	3.69 × 10^−4^	GF
*ERBB3*	4.51	2.49	−0.86	3.69 × 10^−4^	RTK
*DUSP1*	7.06	12.31	0.80	1.13 × 10^−2^	MKP
*DUSP4*	5.89	4.15	−0.50	4.86 × 10^−2^	MKP
*DUSP16*	3.60	8.88	1.30	3.69 × 10^−4^	MKP
*RASA1*	236.29	281.47	0.25	3.69 × 10^−4^	P120GAP
*SOS1*	59.25	56.23	−0.08	4.87 × 10^−2^	SOS
*SOS2*	45.24	40.65	−0.15	3.89 × 10^−2^	SOS
*HRAS*	70.84	52.08	−0.44	3.69 × 10^−4^	RAS
*NRAS*	26.96	31.96	0.25	2.74 × 10^−3^	RAS
*KRAS*	74.05	92.48	0.32	4.08 × 10^−3^	RAS
*RAPGEF2*	29.70	17.79	−0.74	2.49 × 10^−2^	CNRASGEF
*PRKCB*	6.42	2.70	−1.25	4.09 × 10^−2^	PCK
*PRKACB*	12.03	19.53	0.70	2.74 × 10^−3^	PKA
*RAP1B*	297.73	313.97	0.08	3.61 × 10^−2^	RAP1
*RAP1A*	41.91	31.19	−0.43	3.69 × 10^−4^	RAP1
*RAF1*	23.14	33.12	0.52	2.02 × 10^−3^	RAF1
*MAPK1*	107.60	132.85	0.30	3.69 × 10^−4^	ERK
*ELK1*	1.40	3.27	1.22	2.02 × 10^−3^	ELK-1
*ATF4*	179.33	197.24	0.14	6.66 × 10^−3^	CREB
*SRF*	20.37	14.62	−0.48	3.69 × 10^−4^	SRF
*TAB2*	1011.99	1152.61	0.19	3.69 × 10^−4^	TAB2
*PPM1B*	23.61	16.21	−0.54	3.69 × 10^−4^	PP2CB
*MAP3K7*	199.57	262.58	0.40	3.69 × 10^−4^	TAK1
*IKBKB*	4.45	9.20	1.05	6.91 × 10^−4^	IKK

**Table 3:** Statistically significant genes involved in KEGG JNK and p38 MAP kinase pathway with their expression levels in CTR-NSC-34 and VitE-NSC-34, the Fold change computed as the Log_2_(VitE-NSC-34—CTR-NSC-34), the q value and the name of the gene product in KEGG. The values are rounded to the second decimal digit.

Gene	Expression Level CTR-NSC-34	Expression Level VitE-NSC-34	Fold Change	*q* Value	KEGG
*JUND*	3.41	5.15	0.59	2.69 × 10^−2^	JUND
*MAPK9*	118.96	106.30	−0.16	3.44 × 10^−2^	JNK
*MAP2K4*	47.27	77.30	0.71	3.69 × 10^−4^	MKK4
*MAP4K4*	164.30	174.25	0.09	1.78 × 10^−3^	HGK
*CDC42*	323.84	394.04	0.28	3.69 × 10^−4^	CDC42
*MAP3K5*	8.91	16.09	0.85	9.75 × 10^−4^	ASK1
*MAPK11*	3.34	1.83	−0.87	3.24 × 10^−2^	P38
*MAPK14*	40.49	25.46	−0.67	1.11 × 10^−2^	P38
*AKT1*	110.17	136.09	0.31	3.69 × 10^−4^	AKT
*AKT3*	288.28	314.60	0.13	2.25 × 10^−3^	AKT
*DUSP4*	5.89	4.15	−0.50	4.86 × 10^−2^	MKP
*DUSP16*	3.60	8.88	1.30	3.69 × 10^−4^	MKP

**Table 4:** Genes distribution in MAP kinase pathways.

Pathway	Total Number of Genes	Upregulated Genes	Downregulated Genes
Classical MAPK pathway	34 (74%)	16 (47%)	18 (53%)
JNK and p38 MAPK pathway	12 (26%)	8 (67%)	4 (33%)

**Table 5:** Statistically significant genes involved in Mitophagy - Animal KEGG pathway with their expression levels in CTR-NSC-34 and VitE-NSC-34, the Fold change computed as the Log_2_(VitE-NSC-34—CTR-NSC-34), the q value and the name of the gene product in KEGG. The values are rounded to the second decimal digit.

Gene	Expression Level CTR-NSC-34	Expression Level VitE-NSC-34	Fold Change	*q* Value	KEGG
*MAPK9*	118.96	106.30	−0.16	3.44 × 10^−2^	JNK
*ATF4*	179.33	197.24	0.14	6.66 × 10^−3^	ATF4
*MFN2*	74.11	54.96	−0.43	2.02 × 10^−3^	MFN2
*UBB*	6430.17	4355.22	−0.56	3.69 × 10^−4^	UB
*RHOT2*	6.52	1.25	−2.38	1.52 × 10^−3^	MIRO
*BECN1*	248.55	208.86	−0.25	5.27 × 10^−3^	BECLIN1
*USP15*	58.66	69.67	0.25	3.69 × 10^−4^	USP15
*ATG5*	59.74	52.90	−0.18	2.14 × 10^−2^	ATG5
*TAX1BP1*	62.39	31.39	−0.99	3.69 × 10^−4^	TAX1BP1
*SQSTM1*	103.55	160.22	0.63	3.69 × 10^−4^	P62
*NBR1*	52.32	34.50	−0.60	9.60 × 10^−3^	NBR1
*GABARAPL1*	28.21	34.05	0.27	2.71 × 10^−2^	LC3
*RAB7*	258.35	220.73	−0.23	3.69 × 10^−4^	RAB7
